# Modification of Intestinal Flora Can Improve Host Metabolism and Alleviate the Damage Caused by Chronic Hypoxia

**DOI:** 10.3390/cimb46110756

**Published:** 2024-11-10

**Authors:** Zheng Chen, Yang Liao, Shatuo Chai, Yingkui Yang, Qin Ga, Rili Ge, Shuxiang Wang, Shujie Liu

**Affiliations:** 1 Ministry of Agriculture and Rural Affairs Key Laboratory of Animal Nutrition and Forage-Feed of Grazing Yak and Tibetan Sheep in Qinghai-Tibetan Plateau, Key Laboratory of Plateau Grazing Animal Nutrition and Feed Science of Qinghai Province, Yak Engineering Technology Research Center of Qinghai Province, Qinghai Academy of Animal Husbandry and Veterinary Sciences, Qinghai University, Xining 810016, China; czcjsno1@163.com (Z.C.); liaoliaoya25@163.com (Y.L.); xmsykxyqhdx@163.com (S.L.); 2 Research Center for High Altitude Medicine, Qinghai University, Xining 810001, China

**Keywords:** hypoxic pulmonary hypertension, gut microbiota, feed conversion ratio, metabolome, hypoxic and hypobaric exposure

## Abstract

Prolonged exposure to hypoxic conditions can lead to reduced appetite, stunted growth, systemic inflammation, and pulmonary hypertension. Previous studies have indicated a correlation between gut dysbiosis and the development of hypoxia-related hazards. We designed an experiment to investigate the effect of microbiota on mitigating hypoxic damage. Gut microbiota from high-altitude-adapted species *(Ochotona curzoniae)* were transplanted into Sprague Dawley (SD) rats, which were then housed in a simulated 6000 m altitude environment for 30 days. After the experiment, we conducted analyses on average daily weight gain (ADG), feed conversion ratio (FCR), mean pulmonary artery pressure (mPAP), gut flora, and fecal metabolism. The results demonstrated that the ADG in the transplantation group (2.98 ± 0.17 g) was significantly higher than in the control groups (2.68 ± 0.19 g and 2.26 ± 0.13 g) (*p* < 0.05). The FCR was reduced in the transplantation group (6.30 ± 0.33 g) compared to the control groups (8.20 ± 1.15 g and 8.83 ± 0.45 g) (*p* < 0.05). The mPAP was decreased in the transplantation group (38.1 ± 1.13 mmHg) compared to the control groups (43.4 ± 1.30 mmHg and 43.5 ± 1.22 mmHg) (*p* < 0.05). Multi-omics analysis revealed that Lachnospiraceae, Desulfovibrionaceae, and specific amino acid metabolic pathways play crucial roles in hypoxia and are associated with both inflammation and nutritional metabolism. This study proposes a novel approach to the treatment of hypoxic pulmonary hypertension and holds potential significance for improving high-altitude developmental potential.

## 1. Introduction

The detrimental effects of hypoxia on humans and animals are well documented, including decreased appetite, stunted growth, metabolic dysregulation, systemic inflammation, pulmonary hypertension, right ventricular hypertrophy, and heart failure [1,2,3]. Many populations and animals residing in high-altitude regions endure the severe consequences of chronic hypoxia, which significantly hinders the development of these areas [4,5,6]. Previous research indicates a correlation between gut dysbiosis and the progression of hypoxic damage. The gut microbiota plays a crucial role in executing essential physiological functions such as digesting food, harvesting energy, and regulating the immune system. Additionally, it impacts host ecology and aids in adaptation to extreme environments [7,8]. For example, Lachnospiraceae and Ruminococcaceae produce short-chain fatty acids (SCFAs) like butyrate, which help maintain gut health, support energy metabolism, and reduce inflammation. The genus Blautia has been found to respond rapidly to high-altitude hypoxia, helping to maintain intestinal health by reducing inflammation and protecting the intestinal barrier. Overall, high-altitude animals typically have a diverse gut microbiota, ensuring that the gut ecosystem remains stable and functional even under extreme conditions. Furthermore, the gut microbiota of different species exhibits convergence, with shared core microbiota playing a significant role in helping these species adapt to high-altitude environments [9]. The therapeutic targeting of bacterial dysbiosis can be achieved using probiotics (live strains of selected bacteria) or prebiotics (food components that modulate the microbiota). The administration of probiotics, prebiotics, and synbiotics has been shown to significantly attenuate cardiac hypertrophy caused by prolonged hypobaric hypoxia exposure. These interventions have also been found to ameliorate gut microbiome shifts, as well as alterations in short-chain fatty acids, bile acids, amino acids, neurotransmitters, and free fatty acids [10,11]. However, further research is needed to fully understand the practical applications of prebiotics and probiotics [12,13].

*Ochotona curzoniae (Plateau pika)*, a small mammal endemic to the Qinghai–Tibet Plateau, is well adapted to the alpine, anoxic, and resource-scarce environment of the plateau, which ranges from 3000 to 5000 m in altitude [14,15]. They inhabit an open and complex environment characterized by extreme climate changes, food scarcity, and exposure to a wide array of environmental microorganisms [16]. Research shows that the composition and function of the gut microbial communities of *Ochotona curzoniae* are crucial for their ability to adapt to extreme climate conditions and thrive in this challenging plateau environment [17,18,19].

During periods of food scarcity, particularly in winter, *Ochotona curzoniae* supplements its diet by consuming yak feces, resulting in increased convergence of the yak and *Ochotona curzoniae* microbiota. This behavior aids *Ochotona curzoniae* in adapting to the high-altitude environment [20]. Inspired by this, we designed an experiment to transplant gut microbiota from high-altitude-adapted species *(Ochotona curzoniae)* to SD rats, establishing a low-pressure, hypoxia adaptation model. We hypothesized that gut microbiota transplantation would induce significant influence in the physiological responses of the rats to low-pressure hypoxia. This study aims to reveal the effects of gut microbiota transplantation from high-altitude animals on the gut microbiota structure, growth performance, and physiological metabolism of rats under low-pressure, hypoxic conditions. The findings will provide new methods and insights for improving hypoxia adaptation and promoting the health of living beings in high-altitude regions.

## 2. Materials and Methods

### 2.1. Animals and Study Design

**Figure 1:**
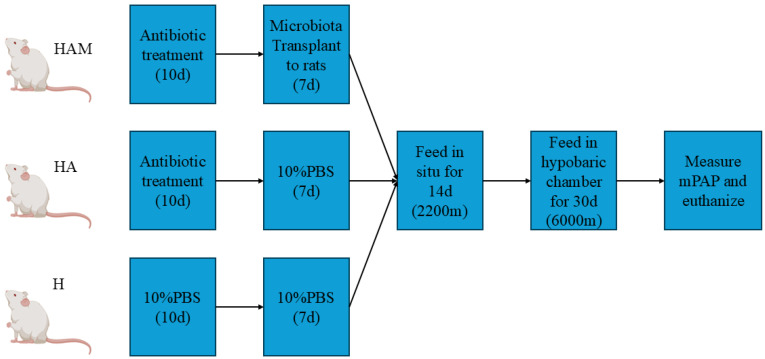
Schematic overview of the experiment.

As seen in Figure 1, a total of 30 SD rats, male, three weeks old, weighing 61 ± 10 g from Beijing Vital River Laboratory Animal Technology Co., Ltd. (Beijing, China), were raised in homogeneous conditions in Xining, Qinghai (2200 m above sea level), fed and watered ad libitum under natural conditions. Weight differences were eliminated at the beginning of the experiments and the rats were randomly assigned to three groups (10 rats per group), as follows: HAO group = Antibiotics + Transplanted microbiota of *Ochotona curzoniae* + Hypoxia; H group =10%PBS + Hypoxia; HA group = Antibiotics + Hypoxia. HAO was the experimental group, HA and H were the control groups. All animal procedures were approved by the Institutional Animal Care and Use Committee of Qinghai University under permission number SL-2021027.

### 2.2. Antibiotic Pretreatment

Following the grouping process, antibiotics were administered as a pretreatment to eliminate the gut microbiome of the rats. The HAO and HA groups were fed an antibiotic (ABx) cocktail (vancomycin 0.5 g L^−1^, ampicillin 1 g L^−1^, neomycin 1 g L^−1^, and metronidazole 1 g L^−1^) for one week before the trial. After this, the HAO and HA groups were subjected to intragastric gavage with 0.5 mL of ABx once daily for three consecutive days. The H group received sterile water for one week prior to being given 0.5 mL of 10% PBS daily for three days via gavage. Upon completion of antibiotic pre-treatment, we promptly collected fecal samples from the H group and ABx group for comparative gut microbiota analysis [21,22].

### 2.3. Fecal Microbiota Donor

Five *Ochotona curzoniae* were live-trapped in Huangyuan, Qinghai Province, at an altitude of 3500 m. An additional five were trapped after three days. Fresh feces from plateau zokors were collected daily, dissolved in 10 mL PBS (1:10) per 1 g, vigorously mixed and homogenized, and centrifuged at 600× *g* for 15 min, and the supernatant was immediately administered orally to the rats in the HAO group [21,22].

### 2.4. Fecal Microbiota Transplantation

After a 24 h antibiotic-free period, the HAO group received 500 µL microbiota suspension once a day by oral gavage for seven days, and the HA and H groups were gavaged with 500 µL of 10% PBS once a day for seven days.

### 2.5. Feeding Experiment

Following the FMT, all rats were fed in situ for two weeks and then transferred to the hypobaric chamber (DYC-300, Guizhou Feng Lei Oxygen Chamber Co., Ltd., Guizhou, China) to be maintained for a period of 30 days. The hypobaric chamber simulated the low-pressure and hypoxic environment at an altitude of 6000 m, with an oxygen concentration of 9.2%.

We entered the chamber once a day to clean, collect information, and observe. At the end of the 30-day feeding period, all rats were weighed, measured for mPAP, and euthanized with urethane.

### 2.6. Hemodynamic Measurements

After 30 days of exposure to hypoxia, 2 rats died in the HAO and HA groups, and 5 rats died in the H group. Urethane (1.0 g/kg) was then used for intraperitoneal anesthesia in the rats. Right heart catheterization was performed through the right jugular vein into the right ventricle and down into the main pulmonary artery to measure mPAP. The inserted catheter was positioned correctly using the waveform shown on the biological function experimental system (BL-420, Tai Meng Technology Co., Ltd., Chengdu, China).

### 2.7. 16S rRDA Sequencing

Fecal samples were collected when the rats were sacrificed, placed in sterile tubes, and stored at −80 °C immediately. The total genomic DNA was extracted using the CTAB/SDS method. The V4 region of the 16S rRNA gene was amplified using barcoded primers for the Illumina platform. The samples were pooled and sequenced with the Illumina NovaSeq platform (NOVOGENE Company Limited, Beijing, China) and 250 bp paired-end reads were generated. Using QIIME and UCHIME, sequences were quality-filtered and trimmed. The operational taxonomic units (OTUs) were chosen based on 97% sequence similarity to the Silva Database. To identify common and unique OTUs among the groups, we evaluated the OTUs using abundance metrics, alpha diversity calculations, Venn diagrams, and other methods. To investigate the differences in community structure among the groups, we performed PCoA dimensionality reduction. Additionally, we used the linear discriminant analysis effect size (LEfSe) statistical analysis to examine the significance of differences in species composition and community structure among the groups.

### 2.8. Fecal Metabolism

Fecal samples were stored at −80 °C and were then sent to NOVOGENE Company Limited (Beijing, China) for metabolite extraction and liquid chromatography–tandem mass spectrometry analysis. Metabolites were annotated using the Kyoto Encyclopedia of Genes and Genomes (KEGG) database, HMDB database, and LIPID Maps Database. A t-test was applied to calculate the statistical significance. The metabolites with VIP > 1.0, FC > 1.2 or FC < 0.833 and *p*-value < 0.05 were considered differential metabolites.

### 2.9. Statistical Analysis

The data were analyzed using SPSS 26.0 statistical software (IBM Co., New York, NY, USA) with a one-way analysis of variance (ANOVA) followed by LSD multiple comparison tests. All groups were compared with each other for every parameter. Values are shown as the means ± standard deviation. Statistical significance was based on *p* < 0.05. The correlation matrix was generated using Spearman’s correlation coefficient performed using the OmicStudio toolsV2.9.1 at https://www.omicstudio.cn/tool/59.3 (accessed on 28 October 2024).

## 3. Results

### 3.1. FMT Improves Weight Gain, Feed Conversion Ratio, and Mean Pulmonary Arterial Pressure in SD Rats

**Figure 2:**
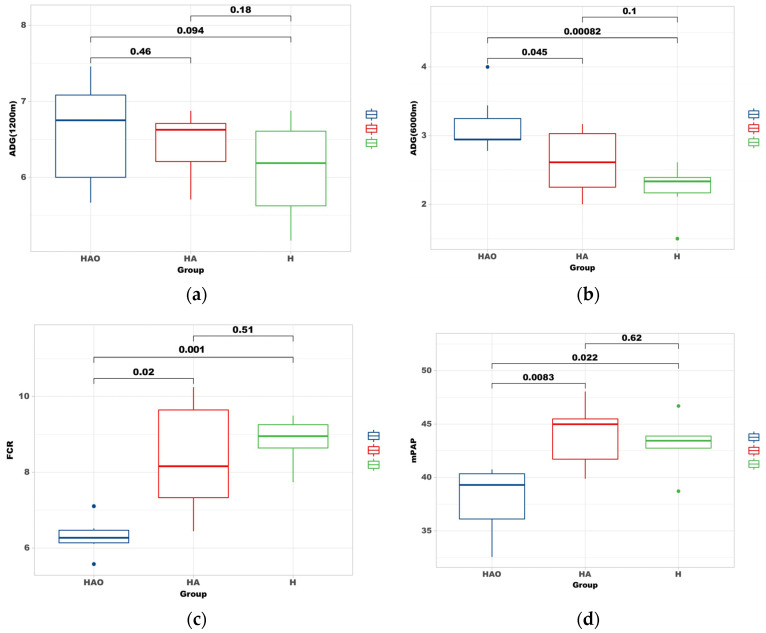
Fecal microbiota transplantation (FMT) modulates weight gain and alleviates pulmonary arterial hypertension. (**a**) Average daily gain (ADG) of the rats at low altitude (2200 m) after FMT. Data are presented as the mean ± standard error of mean (SEM). *p*-values were determined using the *t*-test. (**b**) Average daily gain (ADG) of the rats at high altitude (6000 m) after FMT. (**c**) Feed conversion ratio (FCR) of each group. (**d**) Mean pulmonary arterial pressure (mPAP) in the three groups after FMT.

As shown in Figure 2, the ADG of the rats at low altitude (2200 m) was HAO (6.85 ± 0.21 g) > HA (6.59 ± 0.39 g) > H (6.23 ± 0.23 g). The differences among the groups were not significant (*p* > 0.05) (Figure 2a). At high altitude (6000 m), the ADG was HAO (2.98 ± 0.17 g) > HA (2.68 ± 0.19 g) > H (2.26 ± 0.13 g), with the daily weight gain in the HAO group being significantly higher than that in the HA and H groups (*p* < 0.05). The difference between the HA and H groups was not significant (*p* > 0.05) (Figure 2b). The FCR in the HAO, HA, and H groups was 6.30 ± 0.33 g, 8.20 ± 1.15 g, and 8.83 ± 0.45 g, respectively. It was significantly lower in the HAO group compared to the HA and H groups (*p* < 0.05), with no significant differences between the HA and H groups (*p* > 0.05) (Figure 2c). The mean pulmonary arterial pressure (mPAP) in the HAO, HA, and H groups was 38.1 ± 1.13 mmHg, 43.5 ± 1.22 mmHg, and 43.4 ± 1.30 mmHg, respectively. The mPAP was significantly lower in the HAO group compared to the HA and H groups. The differences in mPAP between the HA and H groups were not significant (Figure 2d).

### 3.2. FMT Contributed to Different Compositions of Gut Microbiota

**Figure 3:**
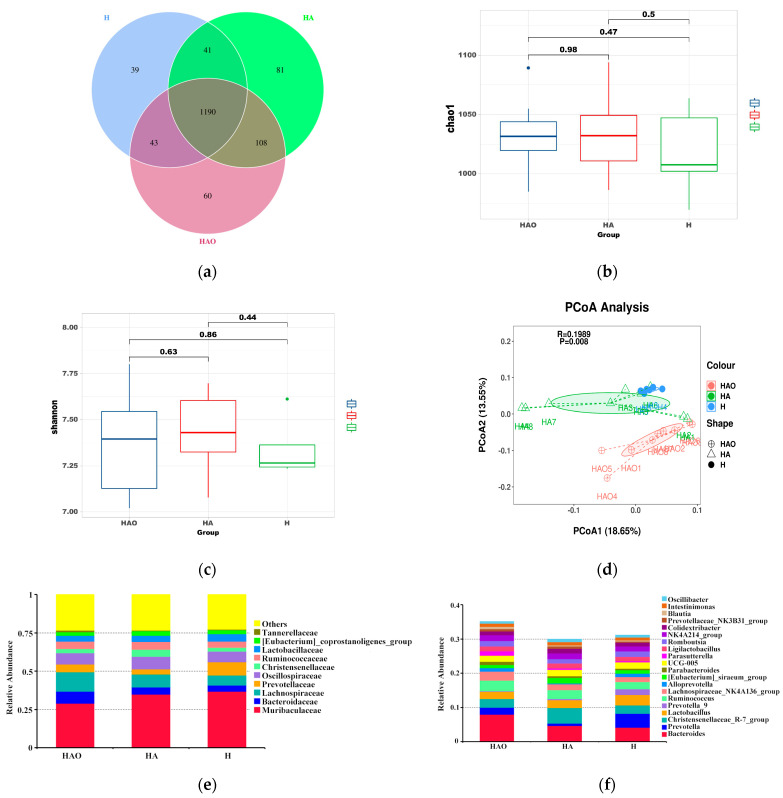
Comparison of gut microbiome between HAO, HA, and H. (**a**) Venn diagram for three groups. (**b**) α-Diversity of different groups as per Chao1. *p*-values were determined using the Wilcoxon test. (**c**) α-Diversity of different groups as per the Shannon index. *p*-values were determined using the Wilcoxon test. (**d**) PCoA analysis based on the unweighted UniFrac distance was performed to visually explore the similarity and variations between the samples’ microbial composition. The percentages in parentheses refer to the proportions of variation explained by each ordination axis. Average relative abundances of dominant bacterial family level (**e**) and genus level (**f**).

To elucidate the gut microbial profiles, we conducted 16S rRNA analyses on the fecal samples. The gut microbial profiles of HAO, HA, and H rats were evaluated using metataxonomic methods. After binning the sequences into operational taxonomic units (OTUs, i.e., groups of sequences sharing a minimum of 97% nucleotide identity), a total of 1562 different OTUs were initially detected across all groups, with 60 of these OTUs detected exclusively in the HAO group; 81 and 39 of these OTUs were only in the HA or H group, respectively (Figure 3a). An alpha diversity analysis showed that the Chao1 was not significantly different between the three groups (*p* > 0.05), nor was the Shannon index (Figure 3b,c). Principal coordinate analysis based on unweighted UniFrac distance yielded dispersed data points on the plots of all groups, implying significant microbial differences in the guts of all groups (*p* < 0.05) (Figure 3d).

The relative abundance of species at the phylum level in each group is shown in Figure S1. The two most abundant species in the three groups were Bacteroidetes and Firmicutes. At the family level, the predominant bacteria were Muribaculaceae, Bacteroidaceae, and Lachnospiraceae and Prevotellaceae (Figure 3e). At the genus level, the top 20 genera were displayed. These were mainly Bacteroides, Prevotella, Christensenellaceae_R-7_group, Lactobacillus and Ruminococcus, and g_Lachnospiraceae_NK4A136_group (Figure 3f).

**Figure 4:**
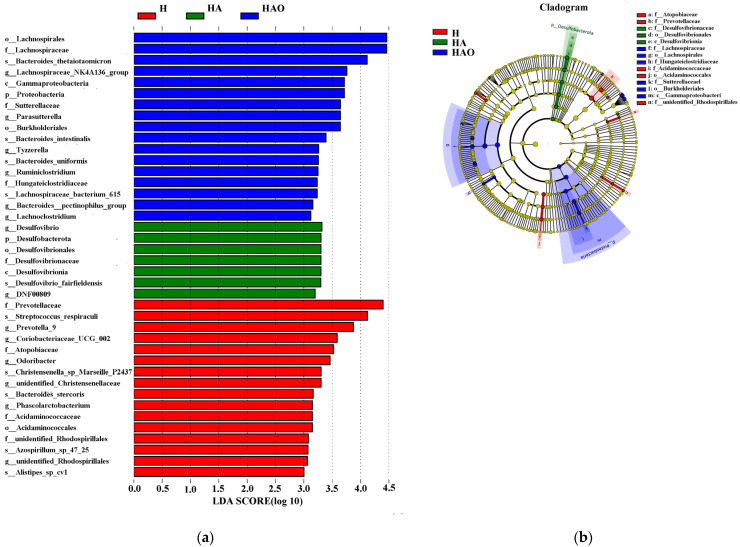
Linear discriminate analysis effect size (LEfSe) was performed to determine the difference in abundance; the threshold of LDA score was 3.0. (**a**) LDA value distribution histogram. (**b**) Evolutionary branch diagram.

Differentially abundant fecal bacterial taxa were further identified using linear discriminant analysis effect size. o_Lachnospirales, f_Lachnospiraceae, and g_Lachnospiraceae_NK4A136_group were abundant in the HAO group, g_Desulfovbrio, f_Desulfovibrionaceae were abundant in the HA group, and f_Prevotellaceae and g_Prevotella_9 were abundant in the H group. Thus, there were remarkable differences in the microbial composition among these groups (Figure 4).

### 3.3. Differential Gut Microbiota Induces Differences in Fecal Metabolites

**Figure 5:**
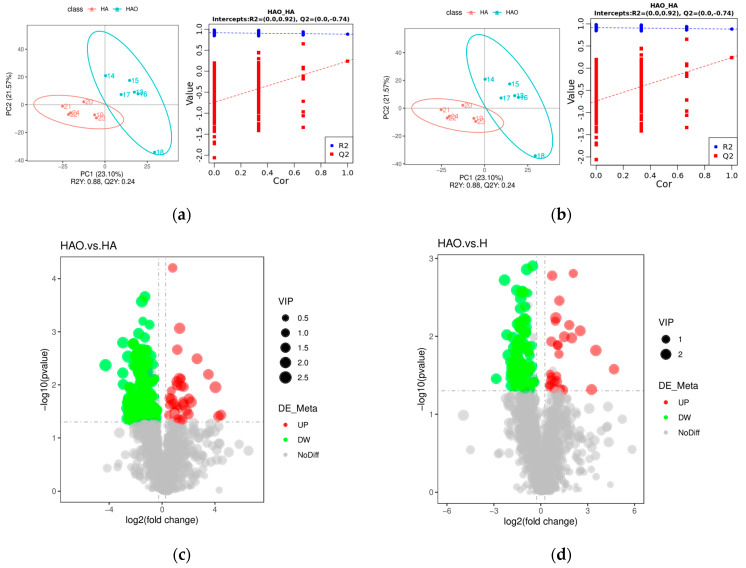
Effect of FMT on the fecal metabolome. (**a**) PLS-DA of fecal metabolites in HAO vs. HA groups. (**b**) PLS-DA of fecal metabolites in HAO vs. HA groups. (**c**) Volcano plot of differential metabolites in HAO vs. H groups. (**d**) Volcano plot of differential metabolites in HAO vs. H groups.

The gut microbiota plays a crucial role in nutrient digestion and absorption, thereby impacting metabolism. In this study, we delved into the effects of FMT on the fecal metabolome using liquid chromatography–tandem mass spectrometry and explored the correlation between metabolites and the gut microbiota. Notably, HAO exhibited distinct metabolites compared to those of HA and H. These differences are visually represented in the partial least squares discriminant analysis plot, illustrating variations in metabolite composition between HAO and HA, as well as between HAO and H (Figure 5a,b).

We employed the following criteria to identify significantly altered metabolites: VIP > 1.0, FC > 1.2 or FC < 0.833, and *p*-value < 0.05. Our analysis revealed 220 and 119 significantly altered metabolites (positive and negative modes) in the HAO vs. HA and HAO vs. H comparisons, respectively. Among these, 35 and 28 metabolites were significantly upregulated in HAO vs. HA and HAO vs. H, respectively, while 185 and 91 metabolites were significantly downregulated (Figure 5c,d). The functions of these metabolites were determined using the KEGG pathway analysis. We analyzed 20 KEGG enrichment pathways, as shown in Figure S2a,b. The results indicated that, in terms of fecal metabolism, the pathways of cysteine and methionine metabolism; biosynthesis of amino acids; aminoacyl-tRNA biosynthesis; glycine, serine, and threonine metabolism; phenylalanine, tyrosine, and tryptophan biosynthesis; C5-branched dibasic acid metabolism; glucosinolate biosynthesis; protein digestion and absorption; methane metabolism; microbial metabolism in diverse environments; and mineral absorption were significantly different between the HAO and HA groups (*p* < 0.05) (Figure S2a). Compared to the H group, the HAO group showed significant differences in methane metabolism, protein digestion and absorption, sphingolipid metabolism, cysteine and methionine metabolism, beta-alanine metabolism, C5-branched dibasic acid metabolism, vitamin B6 metabolism, aminoacyl-tRNA biosynthesis, sphingolipid signaling pathway, and metabolic pathways (*p* < 0.05) (Figure S2b). The relative concentrations of fecal metabolites in the HAO, HA, and H groups were visualized using a heatmap (Figure S3a,b). It showed significant differences in fecal metabolic expression patterns between the HAO group and the HA and H groups.

**Figure 6:**
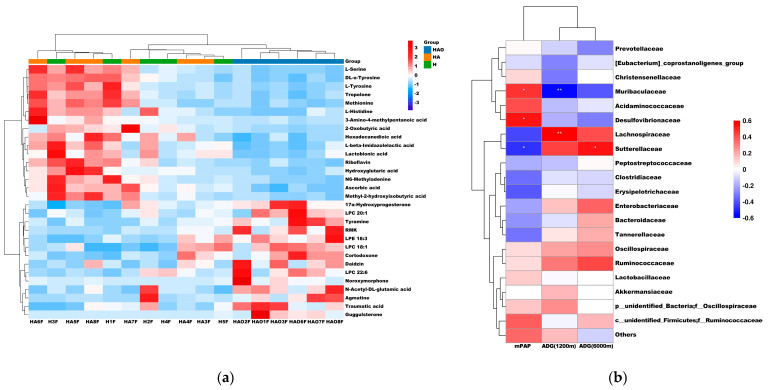
Heatmap of fecal metabolites and heatmap of Spearman correlation. (**a**) Heatmap of 30 metabolites in HAO vs. HA vs. H groups. (**b**) Spearman correlation between intestinal microbiota and mPAP, ADG (2200 m), and ADG (6000 m). Purple denotes a positive correlation; green denotes a negative correlation. The color intensity is proportional to the strength of the Spearman correlation. * *p* ≤ 0.05, ** *p* ≤ 0.01.

We selected 30 significantly different metabolites, which are listed and clustered in Figure 6a. The metabolites enriched in the three groups were significantly different. Metabolites such as agmatine, traumatic acid, LPC 20:1, LPE 18:3, LPC 18:1, and LPC 22:6 are enriched in HAO. L-Serine, DL-O-Tyrosine, L-Tyrosine, tyrosine, methionine, and L-Histidine are enriched in HA and H, particularly in HA. As shown in Figure 6b, we conducted Spearman correlation analysis to examine the relationship between fecal microbes and mPAP, ADG (2200 m), and ADG (6000 m). The results revealed a positive correlation between mPAP and Muribaculaceae and Desulfovbrionaceae, and a negative correlation with Lachnospiraceae and Sutterellaceae. Furthermore, we observed a positive correlation between ADG (6000 m) and Lachnospiraceae, and a negative correlation with Muribaculaceae. Additionally, ADG (2200 m) exhibited a positive correlation with Sutterellaceae.

## 4. Discussion

The importance of the gut microbiota in influencing health and susceptibility to disease is gaining recognition. Weight gain is a crucial indicator of animal growth and development. In this study, under prolonged hypoxic conditions, there were significant differences in weight gain among the rats. The HAO group exhibited superior weight gain and FCR compared to the other groups, demonstrating that microbiota transplantation improved the growth and development of rats in hypoxic environments. Additionally, the mPAP results indicated that the HAO group experienced the least physiological negative impact and hypoxic damage, further confirming that the restructured gut microbiota helped the rats better adapt to hypoxic conditions.

Desulfovibrionaceae, a lipopolysaccharide-producing bacterium, has been implicated in the induction of inflammation and metabolic disorders [23,24], potentially serving as a significant contributor to pulmonary arterial hypertension. Phospholipid metabolites from Desulfovibrio within Desulfovibrionaceae have been identified in the intestinal epithelial CD1d, leading to the proliferation of IL-17A-producing γδ T cells in hypoxic conditions, exacerbating intestinal injury [25]. Notably, the 16s rRNA analysis revealed the lowest levels of Desulfovibrionaceae and Desulfovibrionaceae in the HAO group. Conversely, Lachnospiraceae, known for its production of short-chain fatty acids and conversion of primary to secondary bile acids, plays a crucial role in host–microbe interactions, providing a spectrum of beneficial effects for the host in terms of metabolism and immune regulation, thereby enhancing resistance against intestinal pathogens [26,27]. A reduction in the abundance of Lachnospiraceae may have adverse health implications due to the loss of its multifaceted beneficial functions. Notably, it has been associated with altitude-related cardiac hypertrophy and pulmonary arterial hypertension [13,28]. In addition to these identified bacteria, numerous unknown species are yet to be characterized, necessitating further research to elucidate their roles.

In our experiment, we concentrated on the fecal metabolome to explore the impact of FMT on gut metabolism. While the specific role of the metabolites we identified in hypoxic pulmonary hypertension requires further validation, it is evident that FMT enhanced the metabolic status of the rats, improved their digestive and absorptive capacity, and elevated their overall health. Intestinal microflora have the capability to synthesize and release specific metabolites, which exert a crucial role in regulating various physiological functions in the host. Further investigation into the specific metabolites and their impact on hypoxic pulmonary hypertension could provide valuable insights into the potential mechanisms underlying the observed improvements in the rats’ health.

Amino acids constitute a fundamental class of bioactive macromolecules that play a pivotal role in the construction of biological organisms, serving as essential building blocks for cells and facilitating tissue repair [29]. The metabolic pathway of amino acids is a prerequisite for a myriad of other metabolic processes [30]. The analysis of differential metabolites revealed a substantial presence of amino acid substances in HA and H, including L-Serine, DL-O-Tyrosine, L-Tyrosine, Methionine, and L-Histidine. A comparative analysis of HAO with HA and H further underscored significant disparities in various amino acid metabolic pathways. Intestinal amino acid metabolism is known to be particularly responsive to environmental stress [31], potentially leading to disruptions in amino acid metabolism due to exposure to low pressure and hypoxia. Research has demonstrated the capacity of intestinal microorganisms to influence the host’s intestinal homeostasis, primarily through metabolic pathways such as amino acid metabolism [32]. Furthermore, disorders in amino acid metabolism have been associated with increased intestinal permeability and inflammatory reactions [33], while post-fecal bacterial transplantation, HAO, has been observed to modulate intestinal metabolism, contributing to the maintenance of intestinal homeostasis and nutritional metabolism to a certain extent. To date, there is a limited amount of literature on the implications of alterations in the intestinal environment on the host’s physiological metabolism [34], and this speculation necessitates extensive experimental validation.

The gut microbiota of rats is susceptible to dysbiosis under hypoxic and hypobaric exposure [35]. An imbalance in intestinal flora can alter the intestinal permeability of rats, resulting in fewer mucin-producing goblet cells, shortened villus lengths, and increased intestinal fibrosis and muscular tissue. For example, a reduction in Lachnospiraceae can lead to decreased production of SCFAs, which can alter gut epithelial cells and increase intestinal permeability. An increase in gut permeability allows commensal bacteria to translocate from the enteric cavity into circulation, promoting the generation of peripheral blood bacterial products, which may include endotoxins [36]. Gut dysbiosis can also lead to the high production of inflammatory substances, exacerbating pulmonary hypertension. In this study, the results demonstrated a significant correlation between pulmonary hypertension and the presence of Lachnospiraceae and Desulfovibrionaceae. Desulfovibrionaceae, producers of lipopolysaccharides, have been linked to inflammation and metabolic dysregulation. Conversely, Lachnospiraceae, as producers of short-chain fatty acids, exert influence over the host’s immune functions and inhibit the expression of various inflammatory cytokines [37,38]. Indeed, an increase in Lachnospiraceae and a decrease in Desulfovibrionaceae were observed in the HAO group. Additionally, the HAO group of rats demonstrated the highest feed efficiency, requiring the least amount of feed per unit of body weight. This indicates that, after gut microbiota modification, not only was pulmonary arterial pressure reduced, but the rats’ digestive and metabolic capabilities were also enhanced, leading to improved growth and development.

## 5. Conclusions

This intervention not only enhanced gut flora, such as increasing Lachnospiraceae and decreasing Desulfovibrionaceae, but also improved metabolism, including amino acid metabolism. By altering the microbiota to influence the expression of gut metabolites, FMT mitigated inflammatory responses and improved digestive metabolism. This resulted in enhanced overall health in the rats, promoted their growth and development, and reduced pulmonary arterial pressure. The modification of the gut microbiota significantly enhanced the adaptation of rats to hypoxic environments. However, this study is based on a low-pressure oxygen chamber simulating a high-altitude environment. In reality, high-altitude environments are more complex, involving low pressure, low oxygen levels, low temperatures, and strong ultraviolet radiation. Therefore, further research is needed to determine whether the same effective results can be achieved in practical applications. This study proposes a novel approach to the treatment of hypoxic pulmonary hypertension and provides valuable insights into improving hypoxia adaptation in animals.

## Data Availability

The raw data (16s rRNA Gene Sequencing) that support the findings of this study have been deposited into NCBI under accession number PRJNA767340. The data (Metabolomics and Transcriptome) have been deposited into China National GeneBank DataBase (CNGBdb) under accession number CNP0003641.

## Supplementary Materials

The following supporting information can be downloaded at https://www.mdpi.com/article/10.3390/cimb46110756/s1, Figure S1. Average relative abundances of dominant bacterial phylum level. Figure S2. KEGG enrichment pathways. (a) 20 KEGG enrichment pathways in HAO vs. HA group. (b) 20 KEGG enrichment pathways in HA vs. H group. Figure S3. Heatmap of fecal metabolites. (a) Relative concentrations of fecal metabolites in HAO vs. HA group. (b) Relative concentrations of fecal metabolites in HA vs. H group.
