# Supplementary material for: Modification of Intestinal Flora Can Improve Host Metabolism and Alleviate the Damage Caused by Chronic Hypoxia
Source: Curr Issues Mol Biol. 2024 Nov 10;46(11):12733–45. doi: 10.3390/cimb46110756 (PMC11592817; doi:10.3390/cimb46110756)
Supplement: Supplementary file 1 [file cimb-46-00756-s001.zip › cimb-3258853-supplementary.pdf]

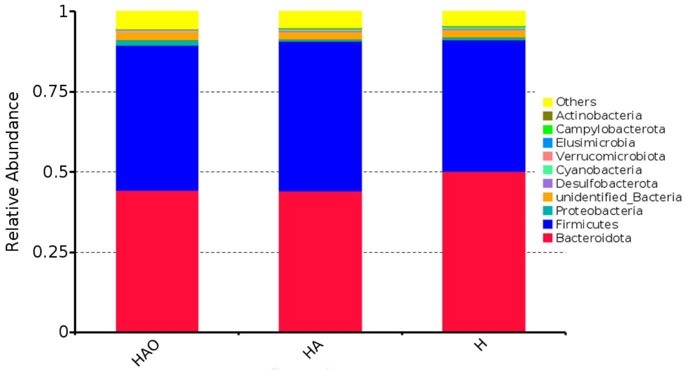

Figure S1. Average relative abundances of dominant bacterial phylum level.

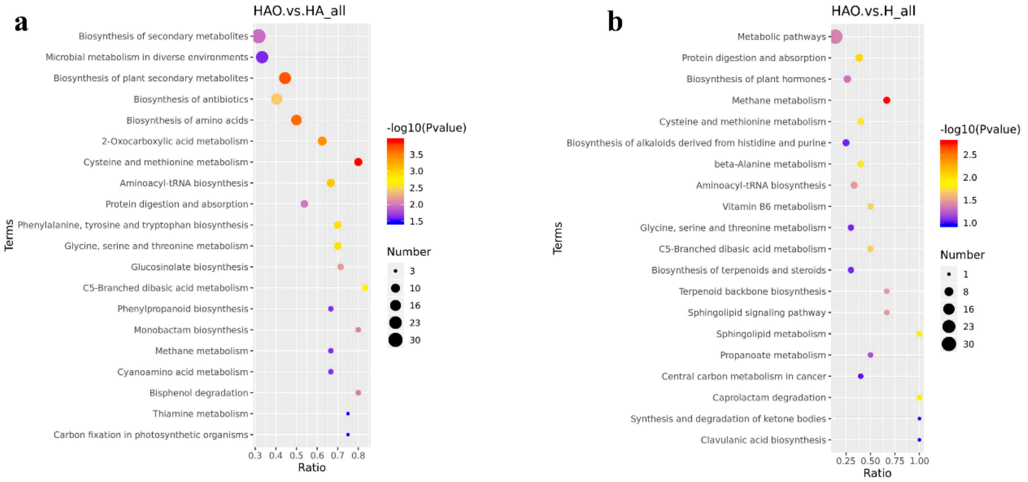

Figure S2. KEGG enrichment pathways. (**a**) 20 KEGG enrichment pathways in HAO vs. HA group. (**b**) 20 KEGG enrichment pathways in HA vs. H group.

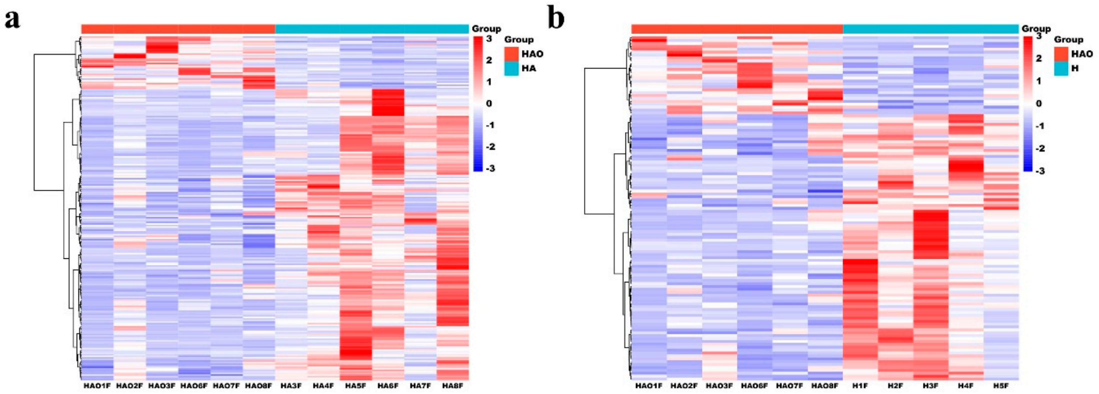

Figure S3. Heatmap of fecal metabolites. (**a**) The relative concentrations of fecal metabolites in HAO vs. HA group. (**b**) The relative concentrations of fecal metabolites in HA vs. H group.
